# A structured framework for effective and responsible generative artificial intelligence chatbot prompt engineering throughout the scientific process: a comprehensive guide for the health and medical researcher

**DOI:** 10.3389/frai.2026.1745928

**Published:** 2026-04-16

**Authors:** Jeremy Y. Ng

**Affiliations:** 1 Institute of General Practice and Interprofessional Care, University Hospital Tübingen, Tübingen, Germany; 2 Robert Bosch Center for Integrative Medicine and Health, Bosch Health Campus, Stuttgart, Germany; 3 Department of Health Research Methods, Evidence, and Impact, Faculty of Health Sciences, McMaster University, Hamilton, ON, Canada; 4 School of Public Health, Faculty of Health, University of Technology Sydney, Sydney, NSW, Australia; 5 Centre for Journalology, Ottawa Methods Centre, Ottawa Hospital Research Institute, Ottawa, ON, Canada

**Keywords:** artificial intelligence, chatbot, GenAI chatbot, generative artificial intelligence, medical research, prompt engineering, research process, scientific process

## Abstract

Generative artificial intelligence (GenAI) chatbots powered by large language models (LLMs) are becoming increasingly integrated into health and medical research workflows, offering researchers new tools to enhance efficiency, support innovation, and assist with knowledge translation. Although their use in health and medical research is expanding rapidly, the practical application of these tools across the broader health and medical research landscape remains complex and evolving. Health and medical researchers often engage with complex study designs, theoretical frameworks, and population needs, all of which require thoughtful, effective and responsible use when involving AI tools. This 10-chapter guide serves as a practical, evidence-informed resource for health and medical researchers to engage effectively and responsibly with GenAI chatbots through the practice of prompt engineering, the design of clear, structured, and purposeful prompts that guide GenAI chatbot outputs. It presents strategies to improve prompt quality and adapt GenAI chatbot interactions to the varied methodological and disciplinary contexts found across health and medical research. The article outlines a structured framework for how GenAI chatbots can be applied throughout the research cycle, including research question development, study design, literature searching, querying for appropriate reporting guidelines and appraisal tools, quantitative and qualitative data analysis, writing and dissemination, and implementation. AI-generated content should be treated as a preliminary draft and must always be reviewed, verified against credible sources, and aligned with disciplinary standards. Risks such as hallucinated content, embedded biases, and ethical challenges are addressed, particularly in sensitive or high-stakes settings. Transparency in AI use and researcher accountability are essential. While GenAI chatbots have the potential to expand access to research support and foster innovation, they cannot replace critical thinking, methodological rigour, or contextual understanding. Instead, they should augment, not replace, human expertise. This guide encourages effective and responsible use of GenAI chatbots and support their thoughtful integration into the health and medical research process.

“The advancements in generative artificial intelligence mark a turning point in human inquiry—research will never again be conceived, conducted, or communicated in the same way—Jeremy Y. Ng.”

## 1 Introduction

Generative artificial intelligence (GenAI) chatbots powered by large language models (LLMs), such as OpenAI’s ChatGPT, Google Gemini (formerly Google Bard), Meta AI, and Microsoft Copilot (formerly Bing Chat), are being increasingly adopted in health and medical research to support and improve a wide range of academic and clinical activities (Ng et al., 2025b; Kwon, 2025; Buess et al., 2025). GenAI chatbots are defined as “chatbots that employ a variety of artificial intelligence technologies, from machine learning comprised of algorithms, features, and data sets that optimize responses over time, to natural language processing and natural language understanding that accurately interpret user questions and match them to specific intents” [International Business Machines (IBM), 2025a]. These tools generate human-like responses to user inputs. As their fluency, accuracy, and versatility continue to advance, GenAI chatbots are emerging as accessible and cost-effective research assistants across many areas of health and medical research (Ng et al., 2025b; Kooli, 2023; Khalifa and Albadawy, 2024).

One of the most important factors influencing the usefulness of GenAI chatbots is prompt engineering. This refers to the practice of constructing clear, targeted, and well-structured instructions to guide the GenAI chatbot’s responses. Well-designed prompts can considerably improve the clarity, accuracy, and relevance of outputs, while vague or poorly framed prompts are more likely to result in generic or misleading responses (White et al., 2023; Giray, 2023). In health and medical research settings, prompt engineering enables researchers to adapt GenAI chatbot interactions for specific domains, support adherence to reporting standards, and reduce the likelihood of bias or misinformation in GenAI-generated content (Heston and Khun, 2023; Wang et al., 2023).

Health and medical researchers often encounter diverse methodological challenges, such as working with complex interventions, evaluating rapidly evolving evidence bases, and navigating discipline-specific terminology (Jacobsen, 2020). These challenges can affect how GenAI chatbots perform, particularly when prompts are not tailored to reflect the context in which the research is being conducted. For example, synthesizing literature in emerging areas of clinical medicine, identifying appropriate study designs in implementation research, or interpreting findings for diverse populations can require prompts that account for these complexities (Ng et al., 2025b). At the same time, GenAI offers unique opportunities, such as supporting the synthesis of literature across multiple languages, enhancing accessibility to underrepresented research areas, and fostering collaboration across disciplines (Akter et al., 2023; Orlando et al., 2024; Ahn, 2024).

Although GenAI chatbots are becoming more widely used in health and medical research (Mabirizi et al., 2025; Biagini, 2025), there has been a lack of specific guidance on how to use them effectively and responsibly throughout the scientific process. While GenAI chatbots remain optional tools in the research process, basic artificial intelligence (AI) literacy and prompt-engineering competence are becoming increasingly relevant for medical researchers akin to other technologies (e.g., calculators, the internet) that have changed the entire way in which research is conducted (OECD, 2020). As GenAI tools grow in accessibility and adoption, familiarity with their capabilities, limitations and ethical implications will likely become part of the core skill set needed to navigate contemporary medical research responsibly. This article aims to fill that gap by providing a practical, evidence-informed guide to prompt engineering for GenAI chatbots in health and medical research more broadly.

## 2 Background: artificial intelligence chatbots and their use in health and medical research

GenAI chatbots powered by LLMs have quickly become prominent tools across practically all research fields, health and medical being no exception. These models are trained on extensive datasets, which enable them to produce coherent, context-sensitive responses to a wide range of user queries in natural language. LLMs use deep learning methods, particularly transformer-based architectures, to understand and generate language by modeling relationships between words, phrases, and ideas [International Business Machines (IBM), 2025b; OpenAI Platform, 2025b; Google AI for Developers, 2025]. As a result, GenAI chatbots can perform complex text-based tasks that have traditionally required human expertise (Singh, 2023). In contrast to conventional software that requires structured commands, these AI systems work through flexible conversational interfaces, allowing researchers to communicate with them in plain language and receive custom responses without requiring specialized technical skills (Dam et al., 2024).

Although the use of GenAI chatbots in health and medical research is expanding rapidly, their application must be approached with care and thoughtful judgment. Researchers now use GenAI chatbots to support nearly all stages of the research process from study design and conceptualization to implementation (Ng et al., 2025b). Some journal editors and peer reviewers are also beginning to use GenAI chatbots for initial screening of manuscripts to assess elements such as structure or adherence to reporting standards (Ng et al., 2025a). Early research suggests that GenAI chatbot-generated outputs may resemble human-written text for basic or routine tasks. However, this does not mean these outputs are automatically reliable. GenAI chatbots may provide incorrect or misleading information, including false references or inaccurate summaries, which highlights the need for careful fact-checking and expert review (Walters and Wilder, 2023; Chen and Chen, 2023; Graf et al., 2024). Researchers should not depend too heavily on these tools and must not replace their own judgment and analytical skills with AI outputs. Instead, GenAI chatbots should be used as supportive tools that enhance productivity and spark new ideas. The final responsibility for ensuring scientific rigour, factual accuracy, and ethical standards remains with the human researcher (Kasani et al., 2024; Dwivedi et al., 2023). In line with this, emerging best practices emphasize the importance of disclosing AI use (and how it was used), acknowledging AI tools that provided assistance, and implementing quality assurance measures when AI is involved in research workflows.

These trends highlight the increasing importance of GenAI chatbots in health and medical research more broadly. As their functionality grows and adoption becomes more widespread, it is crucial for researchers to learn how to engage with them effectively. This includes using prompt engineering strategies that can help guide GenAI chatbots to produce outputs that are more relevant, accurate, and aligned with a given research study’s objectives.

## 3 What is prompt engineering?

Prompt engineering is the deliberate and strategic creation of input queries (or “prompts”) to optimize the outputs generated by GenAI chatbots powered by LLMs. At its core, this practice involves crafting prompts that are clear, targeted, and relevant to the intended task. As LLMs are highly sensitive to how a prompt is phrased, even small adjustments in wording or structure can result in considerably different responses [Google AI for Developers, 2025; International Business Machines (IBM), 2025c; Open AI Platform, 2025a]. In research settings, prompt engineering serves as a critical bridge between researcher expertise and the GenAI chatbot’s capabilities, ensuring outputs are aligned with specific informational needs (White et al., 2023; Giray, 2023; Heston and Khun, 2023; Wang et al., 2023).

There are several commonly used prompt types; the main ones will be described here. Zero-shot prompting asks the GenAI chatbot to perform a task without supplying any examples. While this is appropriate for straightforward tasks, results may vary in specialized contexts [Google AI for Developers, 2025; International Business Machines (IBM), 2025c]. Few-shot prompting includes one or more examples in the query to guide the model’s response, improving consistency and formatting [International Business Machines (IBM), 2025b; OpenAI Platform, 2025b; International Business Machines (IBM), 2025d]. Chain-of-thought prompting encourages the model to reason through problems step by step, which is especially helpful for tasks such as assessing the quality of study methodology or comparing different clinical interventions [Google AI for Developers, 2025; International Business Machines (IBM), 2025e]. Another useful strategy is role-based prompting, where the AI is assigned a specific identity (e.g., “You are an expert in evidence-based medicine” or “Act as the editor of a medical journal”) to shape tone and perspective. These strategies are often most effective when used in combination, tailored to the complexity of the task [Sun et al., 2025; International Business Machines (IBM), 2025f].

Across all these strategies, several key principles form the basis of effective prompt engineering. The Concise, Logical, Explicit, Adaptive, and Reflective (CLEAR) Framework provides a helpful structure for developing high-quality prompts (Lo, 2023). Concise prompts eliminate unnecessary language while maintaining precision, helping the GenAI chatbot stay focused. Avoiding overly broad or verbose inputs improves clarity. Logical prompts follow a well-ordered structure, making it easier for the model to follow a sequence or process, which is particularly helpful in tasks involving stepwise instructions. Explicit prompts specify expectations for output format, content scope, or focus. For example, asking for a summary that emphasizes clinical outcomes rather than general background will yield more targeted responses. Adaptive prompting emphasizes flexibility; users are encouraged to iterate, rephrase, and adjust their inputs to better suit their research topic or disciplinary context. Finally, reflective prompting promotes continuous evaluation and improvement. Researchers should assess AI outputs critically and refine prompts as needed to improve relevance and accuracy. This approach ensures AI is used to support critical thinking and creativity rather than replace them (Open AI Platform, 2025a).

In health and medical research, domain-specific knowledge is essential when crafting prompts. Medical subfields often involve specialized terminology, varied evidence types, and discipline-specific practices that may not be well represented in an AI model’s training data. For instance, prompting an GenAI chatbot to “summarize randomized controlled trials evaluating cognitive behavioural therapy for adolescent anxiety” is far more effective than broadly asking about “mental health therapies for youth,” which may result in generic or unfocused responses. Researchers should define key terms within their prompts, clarify the clinical or research context, and consider how best to frame the request for accurate and relevant responses. Additionally, outputs must be reviewed carefully to ensure that they reflect appropriate scientific frameworks and standards.

As more health and medical researchers incorporate GenAI chatbots into their work, prompt engineering is becoming a core competency. By applying thoughtful, structured, and context-aware prompting techniques, researchers can enhance the performance of GenAI chatbots and minimize risks of misinterpretation, misinformation, or misuse. These skills allow researchers to make the most of AI capabilities while maintaining responsibility for the quality and accuracy of their work.

## 4 Methods used to develop this guide

This guide presents a narrative, evidence-informed synthesis examining the responsible use of generative artificial intelligence (GenAI) chatbots in health and medical research, with particular attention to prompt engineering practices. The methodological approach was informed by principles for high-quality narrative reviews, including those reflected in the Scale for the Assessment of Narrative Review Articles (SANRA) (Baethge et al., 2019).

### 4.1 Literature identification

Relevant literature was identified through structured searches of major academic databases including MEDLINE, EMBASE, Scopus, Web of Science, and Google Scholar, supplemented by targeted searches of grey literature on popular search engines such as Google. Search terms combined keywords related to generative AI and research methodology, including: “generative AI”, “large language models”, “ChatGPT”, “prompt engineering”, “AI in research”, “scientific writing”, and “AI ethics”. Reference lists of relevant articles were also screened to identify additional sources.

### 4.2 Inclusion and selection of sources

Sources were included if they:

- addressed GenAI chatbot use in research or academic contexts,
- discussed prompt engineering, GenAI chatbot-assisted scientific workflows, or methodological implications, or
- provided guidelines, policies, or ethical recommendations for AI or GenAI use in research and scholarly communication.

Both peer-reviewed literature and authoritative grey literature (e.g., guidance from international organizations, editorial policies, and professional bodies) were considered where relevant.

### 4.3 Integration of empirical evidence

In addition to the literature synthesis, the guide draws on insights from four empirical studies conducted by the author, which examined the use of generative AI tools in research and academic workflows. Findings from these studies informed the development of practical recommendations and examples presented throughout the manuscript.

### 4.4 Evidence synthesis

Identified sources were synthesized to develop a practical framework for the responsible use of GenAI across stages of the research lifecycle, including literature exploration, study design, analysis support, and manuscript preparation. Emphasis was placed on identifying effective prompt engineering strategies, potential risks, and safeguards to maintain research rigour, transparency, and ethical integrity. This article is intended as a narrative, evidence-informed synthesis and practical guide rather than a formal review informed by a systematic search. The ultimate goal is to support the effective and responsible use of GenAI chatbots in health and medical research workflows, ultimately contributing to ongoing efforts to improve, rather than compromise, research quality, transparency, and innovation.

## 5 Results

This guide draws on findings that met the aforementioned inclusion criteria. They include four original studies led by the author (as a starting point), comprising three large-scale, international cross-sectional surveys targeting biomedical researchers, university students and postdoctoral fellows, and editors-in-chief of biomedical journals, as well as an audit examining publisher policies on AI (Ng et al., 2025b; Ng et al., 2025a; Ng et al., 2026; Bhavsar et al., 2025). Together, these investigations provided extensive empirical insights into the attitudes, perceptions, and policy approaches toward GenAI chatbots within the biomedical research and publishing sectors. Complementing these studies, guidance from internationally recognized organizations which play foundational roles in advancing high-quality, evidence-informed decision-making by developing rigorous research standards, synthesizing reliable evidence, promoting methodological excellence, and supporting the global translation of scientific knowledge into policy, practice, and education [including Campbell Collaboration, 2025; Cochrane, 2025; Collaboration for Environmental Evidence, 2025; Joanna Briggs Institute (JBI), 2025a; National Academy of Medicine, 2025; National Institute for Health and Care Excellence (NICE), 2024; The Royal Society, 2024; United Nations Educational, Scientific and Cultural Organization, 2022; World Health Organization (WHO), 2025], in addition to guidance from leading organizations that develop international standards for scholarly communication by providing guidance on publication ethics, best editorial practices, authorship responsibilities, and the responsible use of emerging technologies in academic research and publishing [including Committee on Publication Ethics (COPE), 2025; Council of Science Editors (CSE), 2023; European Association of Science Editors (EASE), 2025; The International Association of Scientific, Technical, and Medical Publishers (STM), 2025; International Committee of Medical Journal Editors (ICMJE), 2025, and the World Association of Medical Editors (WAME) (Zielinski et al., 2023)], were reviewed to ensure alignment with current standards around transparency, human oversight, and ethical use of AI tools in academic research and publishing. Additionally, both academic and grey literature on the topics of health and medical sciences, AI, and ethics were searched to identify further recommendations relevant to the responsible integration of GenAI chatbots into research practices.

## 6 Prompt engineering strategies for health and medical researchers

Using GenAI chatbots effectively in health and medical research requires more than simply entering a question. Success depends on carefully constructed prompts that are suited to the complexity of the research task at hand. Prompt engineering refers to the process of developing inputs that guide GenAI chatbots to generate accurate, relevant, and context-aware outputs [Giray, 2023; International Business Machines (IBM), 2025c; Open AI Platform, 2025a; Ekin, 2023; Ray, 2023; Sarkar et al., 2025]. Given the diverse range of research topics and disciplines in health and medical sciences, researchers must use prompt engineering strategies that strengthen the usefulness and trustworthiness of GenAI chatbot interactions.

### 6.1 Establishing context

In health and medical research, clearly defining the context of a prompt is essential to help the GenAI chatbot interpret the request appropriately. Different specialties and clinical areas often use domain-specific terminology, theoretical models, and outcome measures that differ from one another. Prompts should introduce key terms, clarify the subject of interest, and explain how it relates to the broader research goal. For example, rather than saying, “Summarize this trial” after pasting in details of a randomized controlled trial, a health and medical researcher might write, “Summarize this randomized controlled trial evaluating the impact of a cognitive behavioural therapy program on adolescents with generalized anxiety disorder.” Providing this level of detail minimizes misinterpretation and improves the relevance and specificity of the GenAI chatbot’s response.

### 6.2 Structuring the prompt

The way a prompt is structured plays a major role in shaping the GenAI chatbot’s output. Organizing complex tasks into clear, step-by-step instructions typically produces more coherent and useful responses [Giray, 2023; International Business Machines (IBM), 2025c; Open AI Platform, 2025a; Ekin, 2023; Ray, 2023; Sarkar et al., 2025]. For instance, when seeking assistance drafting a manuscript background section, a researcher might use the following format: “(1) Provide an overview of the clinical condition; (2) Describe how the intervention is used in practice; (3) Summarize current evidence from systematic reviews.” Including numbered steps or bullet points creates a clear sequence the GenAI chatbot can follow, especially for multi-component tasks. Specifying formatting preferences, such as requesting section headings, tables, or references, can also help align GenAI chatbot-generated content with scientific writing norms. This is especially valuable when drafting content for academic publication or professional communication.

### 6.3 Iterative refinement

Prompt engineering is inherently iterative. It is uncommon for a first attempt to yield a fully satisfactory response, particularly for complex or nuanced health and medical topics. Researchers should review GenAI chatbot outputs critically and refine their prompts accordingly. If a response lacks specificity, omits important details, or includes questionable claims, follow-up prompts can request clarification, elaboration, or a revised version [Giray, 2023; International Business Machines (IBM), 2025c; Open AI Platform, 2025a; Ekin, 2023; Ray, 2023; Sarkar et al., 2025]. For example, if a GenAI chatbot gives a general description of “inflammation”, the user might follow up with, “Can you explain this concept using analogies that would be understandable to patients without medical training?” This process not only improves the output but also deepens the researcher’s understanding of how to communicate with and benefit from AI systems.

## 7 GenAI chatbot use across the health research lifecycle: a structured framework

While existing literature has largely examined GenAI chatbots in relation to discrete tasks such as drafting text or summarizing literature, their potential role in research practice is broader and extends across multiple stages of the research lifecycle. Framing GenAI chatbot use in relation to the structure of the research process provides a useful way to understand both the opportunities and the limitations of these systems. In this chapter of the guide, a structured framework is proposed that situates GenAI chatbot-assisted activities across key phases of the health and medical research workflow. Within this framework, prompt engineering functions as the central mechanism through which researchers shape model outputs, while methodological safeguards and critical evaluation remain essential to maintaining research rigour and transparency.

### 7.1 Use cases in health and medical research

GenAI chatbots are increasingly being used across the health and medical research process to support a variety of tasks, but their use must always be guided by critical thinking and professional judgment (Khlaif et al., 2023). Early in a project, researchers may use GenAI chatbots to help brainstorm research questions, refine study designs, or draft conceptual frameworks. During literature reviews, they can assist in generating keywords, identifying related concepts, and summarizing abstracts. GenAI chatbots may also introduce researchers to unfamiliar study designs or suggest appropriate reporting guidelines and critical appraisal tools, although final decisions must rest with the researcher’s expertise. In quantitative research, GenAI chatbots may provide explanations of statistical methods, recommend software, or generate code for statistical software. For qualitative work, they can support the development of interview questions or offer example coding frameworks based on known theoretical approaches. GenAI chatbots may also help draft or revise research outputs, including manuscripts, conference abstracts, plain-language summaries, and multilingual content for public engagement. They are increasingly used to support dissemination and implementation efforts, such as tailoring research summaries for policy audiences or suggesting strategies for translating findings into practice (Ng et al., 2025b). However, across all these use cases, AI-generated content should be treated as a starting point only and never a finished product. Every output must be critically reviewed, fact-checked, and tailored to the specific context in which it will be used (Khlaif et al., 2023). The following sections provide detailed examples of how prompt engineering can enhance each stage of the health and medical research process. Supplementary File 1 provides example prompts (and their caveats) relevant to each of these subsections.

### 7.2 Research question development and study design

GenAI chatbots can be useful tools in the early stages of health and medical research for developing research questions and identifying suitable study designs. They can support researchers in generating ideas, narrowing broad topics into focused, answerable questions, and exploring study design options based on key components such as population, intervention, comparison, outcomes, and timeframe (Kooli, 2023). For instance, a GenAI chatbot might help transform a general interest in physical activity interventions for hypertension into a specific research question following the Population, Intervention, Comparator, Outcome, Time (PICOT) framework (Gosak et al., 2025). GenAI chatbots can also suggest commonly used methodologies, including randomized controlled trials, cohort studies, and qualitative designs, along with brief explanations of their strengths and limitations. In complex areas of research, such as implementation science or health services research, GenAI chatbots may prompt researchers to consider mixed methods or pragmatic study designs. They may also assist in drafting logic models or conceptual frameworks that link research objectives to theoretical foundations. However, despite these capabilities, it is essential for researchers to apply critical thinking throughout the process. GenAI chatbots may generate ideas that appear reasonable but are methodologically unfeasible, contextually inappropriate, or misaligned with ethical standards. Because AI lacks discipline-specific reasoning, it may also suggest approaches that are impractical or ethically questionable (Kasani et al., 2024; Dwivedi et al., 2023; Lund et al., 2023).

### 7.3 Literature searches for background and narrative reviews

GenAI chatbots can assist researchers in conducting background research in preparation for the writing of narrative reviews or the introduction chapter of a manuscript. They can help generate keywords, propose related concepts, and provide short summaries of relevant topics (Lund et al., 2023). In fields where terminology can be inconsistent or multidisciplinary, such as when studying psychosocial interventions, emerging therapies, or interdisciplinary public health topics, GenAI chatbots may help identify relevant terms and concepts that could otherwise be missed. GenAI chatbots can also summarize abstracts or clarify unfamiliar terminology, improving the researcher’s ability to navigate the literature (Lund et al., 2023; Glickman and Zhang, 2024; Hwang et al., 2024). However, suggestions generated by AI should be treated as preliminary and not as authoritative. GenAI chatbots may provide inaccurate summaries, omit key sources, or fail to capture important contextual or methodological nuances (Gwon et al., 2024). It is therefore essential for researchers to critically evaluate the quality and relevance of GenAI chatbot outputs and verify all findings using peer-reviewed literature, textbooks, or expert-curated databases. AI tools can enhance efficiency and promote creative thinking, but they are not substitutes for rigorous scholarship. When used thoughtfully, GenAI chatbots can complement the research process, but the responsibility for accuracy and interpretive depth lies with the researcher.

### 7.4 Reviews with systematic search components

For structured reviews involving systematic search processes, such as systematic or scoping reviews, GenAI chatbots can provide useful support during the initial planning and preparation stages. They may assist in formulating preliminary research questions, suggesting synonyms and Boolean operators, recommending relevant databases, and guiding the use of controlled vocabularies such as Medical Subject Headings (MeSH) or Emtree terms, for the Medical Literature Analysis and Retrieval System Online (MEDLINE) and Excerpta Medica database (EMBASE) bibliographic databases, respectively (Park et al., 2024). These functions can be particularly beneficial in areas of health and medical research where terminology varies across disciplines and regions. For example, a GenAI chatbot might help a researcher identify different ways an intervention is described in the literature (e.g., synonyms, related terms, or variations in terminology used across disciplines) for a pharmacological treatment applied in both clinical and community settings, or suggest strategies for categorizing interventions within a review protocol.

Despite these advantages, GenAI chatbots are not capable of conducting actual database searches, applying inclusion and exclusion criteria, or meeting formal methodological standards. They are also not a substitute for the expertise of an information specialist when developing and refining search strategies (Gwon et al., 2024; Park et al., 2024). Despite newer models of GenAI chatbots now having live access to indexing systems, they still may generate incomplete references or fabricated citations (Gwon et al., 2024). Researchers should therefore use AI-generated content with caution and validate all outputs by manually reviewing sources and consulting academic librarians or systematic review experts. Critical thinking is essential when interpreting search strategies, selecting studies, and synthesizing results. Overreliance on AI-generated suggestions can weaken the rigour of the review process (Kasani et al., 2024). Used appropriately, GenAI chatbots can help initiate brainstorming and draft early strategies for structured reviews, but the responsibility for conducting a transparent, reproducible, and high-quality review remains with the researcher.

### 7.5 Research methodology, reporting guidelines, and critical appraisal

GenAI chatbots may offer useful support in the areas of research methodology, reporting guidelines, and critical appraisal, although current evidence evaluating their effectiveness in this domain remains limited. In practice, one of the most reliable uses of GenAI chatbots is to help researchers identify relevant methodological frameworks, reporting guidelines, or appraisal tools based on the study’s objectives, design, or research question. GenAI chatbots can be particularly helpful for researchers who are new to reporting guidelines, as they can describe each reporting guideline’s purpose, scope, and core elements in more understandable terms. Commonly used reporting guidelines include the Consolidated Standards of Reporting Trials (CONSORT) for randomized controlled trials (Schulz et al., 2010), the Preferred Reporting Items for Systematic Reviews and Meta-Analyses (PRISMA) for systematic reviews (Page et al., 2021), and the Strengthening the Reporting of Observational Studies in Epidemiology (STROBE) for observational research (Von Elm et al., 2007). In addition to these widely used guidelines, numerous reporting guidelines specific to the use of AI in medical research exist [Kolbinger et al., 2024; Enhancing the Quality and Transparency of Health Research (EQUATOR) Network, 2025a], with many more currently under development [Enhancing the Quality and Transparency of Health Research (EQUATOR) Network, 2025b]. Furthermore, GenAI chatbots may also assist researchers in identifying and navigating other reporting guidelines relevant to different study types (e.g., diagnostic accuracy, qualitative research, or health economic evaluations) as well as various extensions to existing guidelines that address specific methods, populations, or data types [Enhancing the Quality and Transparency of Health Research (EQUATOR) Network, 2025c]. For the assessment of study methodological quality, GenAI chatbots may also recommend relevant appraisal tools such as the Joanna Briggs Institute (JBI) Critical Appraisal Tools [Joanna Briggs Institute (JBI), 2025b], the Critical Appraisal Skills Programme (CASP) checklists [Critical Appraisal Skills Programme (CASP), 2025], or the tools developed by the Oxford Centre for Evidence-Based Medicine (CEBM) [Centre for Evidence-Based Medicine (CEBM), 2025]. While these features can be useful, researchers must have at least a basic understanding of the reporting guidelines or appraisal tools that they are using to critically assess the accuracy and suitability of GenAI chatbot recommendations. AI-generated suggestions should be seen as preliminary aids, not final answers. Researchers must always evaluate the content for accuracy and cross-check it with authoritative, peer-reviewed sources. GenAI chatbots are not replacements for expertise or critical reasoning. Used properly, they can support the methodological aspects of research, but all outputs should be verified and interpreted through the lens of scientific rigour and professional judgment.

### 7.6 Quantitative analysis

In quantitative health and medical research, GenAI chatbots can assist researchers in exploring and understanding statistical techniques and available software tools. For those with limited backgrounds in statistics, GenAI chatbots can suggest commonly used methods, explain foundational concepts, and introduce software options such as R, Python, SPSS, or STATA (OpenAI, 2025). This support can be particularly helpful during the initial planning stages of a project, when users are selecting appropriate tools or learning new approaches. For example, a GenAI chatbot might explain when to apply non-parametric tests like the Mann–Whitney U test or describe how to use generalized linear models for skewed outcome distributions. More experienced users may prompt GenAI chatbots to help generate or troubleshoot code for statistical analyses. A researcher might request R or Python code to perform chi-square tests, clean a dataset with missing values, or visualize repeated measures data from a clinical trial (Ruta et al., 2025; Prandner et al., 2025; Huang et al., 2024). However, caution is required, as errors introduced by AI-generated suggestions early in a project may compound and influence subsequent analyses. Studies have shown that AI-generated code and recommendations can include subtle but important errors, suggest inappropriate statistical tests, or rely on flawed assumptions, particularly when provided with limited context (Huang et al., 2024; Ordak, 2023; Koçak, 2025). Therefore, all AI-generated outputs should be treated as preliminary drafts and carefully reviewed, tested on actual data, and validated using trusted sources such as statistical texts, peer-reviewed methods papers, or consultation with a statistician (Prandner et al., 2025; Huang et al., 2024; Schwarz, 2025). GenAI chatbots are best viewed as supplements that can improve efficiency and access to methods, rather than replacements for domain expertise or critical evaluation.

### 7.7 Qualitative research

Qualitative research is essential for understanding experiences, perspectives, and contexts that cannot be captured through quantitative methods alone. GenAI chatbots can support several stages of qualitative health and medical research, particularly during the planning and design phases (Zhang et al., 2025). For example, researchers can prompt GenAI chatbots to help develop semi-structured interview or focus group guides. By specifying the study population (e.g., adults undergoing physiotherapy for chronic back pain), the intervention (e.g., exercise-based rehabilitation), and the research objective (e.g., exploring patient perceptions of treatment barriers), users can generate customized open-ended questions consistent with qualitative research standards (Gosak et al., 2025; Christou, 2023). During the analysis phase, GenAI chatbots can provide guidance on methodological approaches such as thematic analysis, grounded theory, or framework analysis (Wachinger et al., 2024; Lee et al., 2024; Turobov et al., 2024). A user might ask for example codes for a dataset on patient experiences in telehealth consultations or request a sample coding structure based on a particular theory. It is important to note that GenAI chatbots operate via mechanistic pattern-matching and lack the reflexivity, contextual understanding, and interpretive reasoning that underpin qualitative inquiry. Generated coding suggestions may miss subtle meanings, fail to capture context-dependent nuances, or inadvertently reinforce stereotypes (Wachinger et al., 2024; Lee et al., 2024; Nguyen-Trung, 2025; Bijker et al., 2024; Morgan, 2023). Consequently, AI support should not replace critical engagement with the data, theoretical reasoning, or the researcher’s interpretive insight. Researchers should approach AI outputs as organizational or illustrative aids, while ensuring that all substantive interpretation, ethical considerations, and reflexive analysis remain firmly within the human researcher’s purview (Lee et al., 2024; Sakaguchi et al., 2025).

### 7.8 Writing and dissemination

GenAI chatbots can assist with the writing and dissemination of health and medical research findings across a variety of formats and audiences. However, researchers should proceed with great caution when using GenAI chatbots to draft any portion of a journal manuscript or conference abstract. While AI tools can enhance efficiency and help generate new ideas, researchers must apply critical thinking to evaluate the accuracy, relevance, and scientific integrity of any AI-generated content (Huang and Tan, 2023). A cross-sectional audit of 162 scientific, technical, and medical publishers found that none permitted GenAI chatbots to be listed as authors on submitted manuscripts (Bhavsar et al., 2025). This reflects the principle that authorship implies accountability, which AI tools cannot assume, as they are incapable of ethical, legal, or intellectual responsibility [Committee on Publication Ethics (COPE), 2025; Council of Science Editors (CSE), 2023; European Association of Science Editors (EASE), 2025; The International Association of Scientific, Technical, and Medical Publishers (STM), 2025; International Committee of Medical Journal Editors (ICMJE), 2025; Zielinski et al., 2023; Bird et al., 2020]. GenAI chatbots may be helpful for drafting cover letters, lay summaries, or other content intended for scientific communication and public engagement (Pollesello and Papp, 2023; Hendriks et al., 2025; Valle and Barone, 2024). They can also support the reframing of technical research findings for different audiences by modifying tone, structure, or language (Li et al., 2024; Markowitz, 2024; Faris, 2023). For example, an GenAI chatbot might assist in rewriting a complex explanation of a clinical intervention into a plain-language summary suitable for a community newsletter. In multilingual or intercultural settings, researchers may find GenAI chatbots helpful for translating content or simplifying technical information for broader audiences (Akter et al., 2023; Orlando et al., 2024; Ahn, 2024). GenAI chatbots can also help generate content for social media platforms such as Twitter/X, LinkedIn, or Instagram. For instance, a researcher might prompt the GenAI chatbot to create a thread, caption, or summary post communicating study findings in an accessible and engaging way. This may be especially beneficial for researchers working in low-resource environments or those without institutional communications support. That said, social media presents unique challenges given its short format, high visibility, and potential for misinterpretation (Tandar et al., 2024; Morita et al., 2024). Currently, there is limited evidence guiding the use of GenAI chatbots for social media content creation in research contexts.

Regardless of the communication channel, any content generated by a GenAI chatbot should be treated as a preliminary draft. All content must be reviewed for scientific accuracy, aligned with the original research findings, and carefully edited before submission or publication. When used responsibly, GenAI chatbots can facilitate the writing and dissemination process (Resnik and Hosseini, 2024). However, the researcher remains fully accountable for ensuring the accuracy, clarity, and appropriateness of all final outputs, whether published in academic journals, presented at conferences, or shared with the public. Overreliance on AI-generated material without proper oversight may introduce factual errors, fabricated citations, or inappropriate phrasing, potentially undermining the credibility of the research (Dwivedi et al., 2023; Huang et al., 2024; Hendriks et al., 2025; Loh, 2023; Kacena et al., 2024). Such errors can jeopardize publication and erode trust in both the individual researcher and the broader scientific community (Williamson and Prybutok, 2024).

### 7.9 Implementation

The translation of research findings into practice, whether in clinical care, education, or policy, is a critical step in maximizing its real-world impact. Although AI technologies have been studied in various implementation science contexts, there is limited research specifically examining the role of GenAI chatbots in supporting implementation efforts (Hogg et al., 2023; Gama et al., 2022; Nilsen et al., 2022). GenAI chatbots can contribute to this process by helping researchers generate ideas for implementation strategies, identify potential barriers and facilitators, or tailor interventions to specific settings (Trinkley et al., 2024). For example, a GenAI chatbot may assist in brainstorming how to incorporate a validated behavioural intervention into primary care workflows or how to adapt a mobile health app for use in underserved rural communities. GenAI chatbots may also support the development of implementation plans, suggest theoretical frameworks such as the Consolidated Framework for Implementation Research (CFIR) (Damschroder et al., 2009; Damschroder et al., 2022) or the Reach, Efficacy, Adoption, Implementation, and Maintenance (RE-AIM) Model (Glasgow et al., 1999; Glasgow et al., 2019), and generate logic models that align with key research objectives. In educational contexts, GenAI chatbots may help draft training materials or identify approaches for integrating new evidence into professional development curricula (Ng et al., 2026; Singh et al., 2023). In policy contexts, they can support the creation of summaries or framing strategies that align research findings with decision-makers’ priorities (Reddy, 2024).

Despite these potential benefits, researchers must recognize the limitations of GenAI chatbots in the implementation domain. AI-generated outputs often lack awareness of local context, community dynamics, logistical realities, and institutional constraints, all of which are crucial to successful implementation (Trinkley et al., 2024). For this reason, any suggestions produced by GenAI chatbots must be critically reviewed and refined through consultation with relevant stakeholders, implementation science experts, and contextual evidence. While GenAI chatbots may enhance early planning or ideation, they are not a replacement for the complex, collaborative work required to bring evidence into practice (Trinkley et al., 2024). Used thoughtfully, GenAI chatbots can support the implementation process by helping generate supporting materials or exploring ideas, but the ultimate responsibility for evaluating, adapting, and executing implementation strategies rests with the researcher.

## 8 Structured framework for best practices and recommendations for using artificial intelligence chatbots in health and medical research

Table 1 provides a structured framework for best practices and recommendations for using GenAI chatbots in health and medical research. Additionally, selected AI and GenAI guidance documents which were used, in part, to inform the writing of this guide are provided in Tables 2, 3.

**Table 1:** Structured framework for best practices and recommendations for using GenAI chatbots in research.

Section	Use case	Best practices and recommendations
7.2	Research Question Development and Study Design	Use GenAI chatbots for brainstorming and refining research questions, exploring study designs, and drafting conceptual frameworks. Always verify that the suggestions are methodologically appropriate and contextually aligned. Apply critical thinking to avoid relying on flawed, unethical, or unfeasible recommendations.
7.3	Literature Searches for Background and Narrative Reviews	Use GenAI chatbots to generate keywords, summarize abstracts, and explore unfamiliar concepts. Treat outputs as preliminary and verify them against reliable sources. Be cautious of cultural misrepresentations or oversimplified explanations.
7.4	Reviews with Systematic Search Components	Use GenAI chatbots to plan search strategies, including generating synonyms and database suggestions. Do not rely on GenAI chatbots for executing searches or evaluating study quality. Collaborate with information specialists and validate all GenAI chatbot-generated strategies manually.
7.5	Research Methodology, Reporting Guidelines, and Critical Appraisal	Use GenAI chatbots to identify relevant reporting guidelines (e.g., Consolidated Standards of Reporting Trials (CONSORT), the Preferred Reporting Items for Systematic Reviews and Meta-Analyses (PRISMA), and the Strengthening the Reporting of Observational Studies in Epidemiology (STROBE), among other reporting guidelines and extensions) or appraisal tools (e.g., Joanna Briggs Institute (JBI) Critical Appraisal Tools, the Critical Appraisal Skills Programme’s (CASP) checklists, the Oxford Centre for Evidence-Based Medicine’s (CEBM) critical appraisal tools) and summarize their scope. Researchers should have basic knowledge of these items to assess GenAI chatbot suggestions. Always confirm accuracy with authoritative sources and do not rely on AI for final methodological decisions.
7.6	Quantitative Analysis	Use GenAI chatbots to explore statistical methods or suggest software relevant to the planned analyses. For novice users, treat responses as educational rather than analytical. For more experienced users, GenAI chatbots can generate draft statistical code, but all code and underlying assumptions must be carefully reviewed, tested on real data, and validated against trusted sources. Avoid running AI-generated code without thorough verification, as errors introduced early on can compound and affect subsequent analyses.
7.7	Qualitative Research	Use GenAI chatbots to generate the first draft of interview guides, propose coding frameworks, or explain qualitative methods, all of which should then be reviewed carefully. AI-generated codes, themes, or frameworks should never replace direct engagement with data, reflexive analysis, or theoretical interpretation. Critically assess outputs, avoid overreliance, and ensure alignment with cultural, contextual, and theoretical perspectives.
7.8	Writing and Dissemination	Use GenAI chatbots to draft manuscripts, abstracts, plain language summaries, or social media content with extreme caution. Treat outputs as drafts only. Verify all facts, ensure alignment with journal guidelines, and be transparent about AI use. Overreliance may result in misrepresentation of evidence, which in turn may undermine the credibility of the research, jeopardize publication, and erode trust in both the individual researcher and the broader scientific community.
7.9	Implementation	Use GenAI chatbots for brainstorming implementation strategies, drafting plans, or identifying relevant frameworks (e.g., CFIR, RE-AIM). Critically assess outputs and adapt them with input from stakeholders. AI cannot replace localized, interdisciplinary decision-making.
All	Sharing Study Data/Unpublished Findings with an GenAI Chatbot	In case of uncertainty, do not upload sensitive, identifiable, or unpublished study data into public GenAI chatbots, as this may violate confidentiality agreements, data protection regulations (e.g., GDPR, HIPAA), and ethical approvals. Use institutionally approved, secure environments if AI-assisted analysis is desired, and ensure that any proposed uses have been approved by the local institutional review board.

**Table 2:** Selected AI and GenAI guidance documents from internationally recognized organizations which play foundational roles in advancing high-quality, evidence-informed decision-making by developing rigorous research standards, synthesizing reliable evidence, promoting methodological excellence, and supporting the global translation of scientific knowledge into policy, practice, and education.

Organization (listed alphabetically)	Report/source title	Publication year	URL
Campbell Collaboration	Position Statement on Artificial Intelligence (AI) Use in Evidence Synthesis Across Cochrane, the Campbell Collaboration, JBI and the Collaboration for Environmental Evidence 2025*	2025	doi: https://10.1002/cl2.70074
Cochrane	Position Statement on Artificial Intelligence (AI) Use in Evidence Synthesis Across Cochrane, the Campbell Collaboration, JBI and the Collaboration for Environmental Evidence 2025*	2025	doi: 10.1002/14651858.ED000178
Collaboration for Environmental Evidence (CEE)	Position Statement on Artificial Intelligence (AI) Use in Evidence Synthesis Across Cochrane, the Campbell Collaboration, JBI and the Collaboration for Environmental Evidence 2025*	2025	doi: 10.1186/s13750-025-00374-5
Joanna Briggs Institute (JBI)	Position Statement on Artificial Intelligence (AI) Use in Evidence Synthesis Across Cochrane, the Campbell Collaboration, JBI and the Collaboration for Environmental Evidence 2025*	2025	doi: 10.11124/JBIES-25-00480
National Academy of Medicine (NAM)	Generative Artificial Intelligence in Health and Medicine: Opportunities and Responsibilities for Transformative Innovation	2025	doi: 10.17226/28907
National Institute for Health and Care Excellence (NICE)	Use of AI in Evidence Generation: NICE Position Statement	2024	https://www.nice.org.uk/position-statements/use-of-ai-in-evidence-generation-nice-position-statement
The Royal Society	Science in the Age of AI	2024	https://royalsociety.org/news-resources/projects/science-in-the-age-of-ai/
United Nations Educational, Scientific and Cultural Organization (UNESCO)	Recommendation on the Ethics of Artificial Intelligence	2022	https://digitallibrary.un.org/record/4062376
World Health Organization (WHO)	Ethics and Governance of Artificial Intelligence for Health: Guidance on Large Multi-Modal Models	2025	https://www.who.int/publications/i/item/9789240084759
	Using AI for Rapid Evidence Synthesis: Training Package for Using AI for Rapid Evidence Synthesis	No Year Provided	https://www.emro.who.int/evidence-data-to-policy/training-package/using-ai-for-rapid-evidence-synthesis.html

*Published simultaneously with Cochrane Database of Systematic Reviews, Campbell Systematic Reviews, JBI Evidence Synthesis, and Environmental Evidence. The articles are identical except for minor stylistic and spelling differences in keeping with each journal’s style.

**Table 3:** Selected AI and GenAI guidance documents from leading organizations that develop international standards for scholarly communication by providing guidance on publication ethics, best editorial practices, authorship responsibilities, and the responsible use of emerging technologies in academic research and publishing.

Organization (listed alphabetically)	Report/source title	Publication year	URL
Committee on Publication Ethics (COPE)	Authorship and AI Tools	2023	https://publicationethics.org/guidance/cope-position/authorship-and-ai-tools
Council of Science Editors (CSE)	2.1.15 Use of Artificial Intelligence (AI) in the Work, In: 2.1 Editor Roles and Responsibilities	2023	https://cse.memberclicks.net/2-1-editor-roles-and-responsibilities#UseofArtificialIntelligenceintheWork
European Association of Science Editors (EASE)	Recommendations on the Use of AI in Scholarly Communication	2024	https://ease.org.uk/2024/09/recommendations-on-the-use-of-ai-in-scholarly-communication/
International Association of Scientific, Technical and Medical Publishers (STM)	Recommendations for a Classification of AI Use in Academic Manuscript Preparation	2025	https://stm-assoc.org/document/recommendations-for-a-classification-of-ai-use-in-academic-manuscript-preparation/
International Committee of Medical Journal Editors (ICMJE)	Defining the Role of Authors and Contributors	No Year Provided	https://www.icmje.org/recommendations/browse/roles-and-responsibilities/defining-the-role-of-authors-and-contributors.html
World Association of Medical Editors (WAME)	Chatbots, Generative AI, and Scholarly Manuscripts: WAME Recommendations on Chatbots and Generative Artificial Intelligence in Relation to Scholarly Publications	2023	https://wame.org/page3.php?id=106

## 9 Discussion

### 9.1 Limitations and challenges to artificial intelligence chatbot use in health and medical research

While GenAI chatbots show great potential for supporting health and medical research, their use also presents notable limitations. One of the most pressing concerns is the tendency of large language models to produce hallucinations, plausible sounding but inaccurate or entirely fabricated content (Huang et al., 2025; Teubner et al., 2023). In health and medical research, this can manifest as misrepresented study findings, fictitious references, or misleading summaries of scientific literature (Walters and Wilder, 2023; Chen and Chen, 2023; Graf et al., 2024; Loh, 2023). These issues can compromise research accuracy and contribute to the spread of misinformation if AI-generated outputs are accepted uncritically.

Another important concern is the presence of biases in the training data used to develop GenAI chatbots. Most large language models are trained on publicly available internet text and academic literature, which often reflect dominant perspectives within biomedicine and may underrepresent emerging evidence that challenges longstanding assumptions or reveals shifts in scientific understanding (Cirillo et al., 2020; Chow and Li, 2024; Nazi and Peng, 2024; Fletcher et al., 2021; Faizan et al., 2025). As a result, GenAI chatbots may produce outputs that downplay emerging research areas or reinforce dominant paradigms without accounting for the diversity of viewpoints and evolving evidence bases. For example, an GenAI chatbot may incorrectly label an innovative clinical approach as ineffective due to gaps in mainstream literature, overlooking ongoing trials or emerging consensus that may not have yet been included in its training data.

In addition to technical limitations, the use of generative AI tools introduces important cognitive and epistemological risks for researchers. One such concern is automation bias, a phenomenon in which individuals place excessive trust in outputs generated by automated systems (Bansal et al., 2021; Green and Chen, 2019). Because large language models generate responses that are fluent, coherent, and confidently phrased, users may be inclined to accept these outputs without sufficient scrutiny, particularly when responses appear plausible or align with prior expectations. This tendency can contribute to the uncritical acceptance of incorrect interpretations, fabricated references, or incomplete summaries of evidence (Bansal et al., 2021; Green and Chen, 2019; Haltaufderheide and Ranisch, 2024).

Closely related to automation bias is the issue of epistemic authority. Although GenAI chatbots can produce articulate explanations across a wide range of topics, they do not possess domain expertise, methodological judgment, or the capacity to critically evaluate evidence. Their outputs are generated by identifying statistical patterns in training data rather than by assessing the validity or reliability of scientific claims (Shin, 2025; Bender et al., 2021; Heersmink et al., 2024; Bang et al., 2023). Consequently, the persuasive presentation of AI-generated text may inadvertently influence researcher judgment, potentially shaping interpretations or decisions even when the underlying content is inaccurate or incomplete.

The increasing use of GenAI-assisted reasoning tools may also influence how scientific knowledge is produced. Generative models can assist with early stages of research activity, including brainstorming research questions, exploring conceptual relationships, or summarizing bodies of literature (Dwivedi et al., 2023; Lund et al., 2023). While such capabilities may enhance efficiency and stimulate new ideas, they may also subtly influence the framing of research questions or the direction of inquiry.

The quality of GenAI chatbot responses is also highly influenced by how prompts are written. Poorly designed prompts can lead to vague, irrelevant, or overly generic responses (Giray, 2023; International Business Machines (IBM), 2025c; Open AI Platform, 2025a; Ekin, 2023; Ray, 2023; Sarkar et al., 2025). New users may underestimate the importance of prompt clarity, resulting in inconsistent or misleading outputs. Additionally, many areas of health and medical research require nuanced phrasing to reflect complex theoretical models, clinical practices, or population-specific terminology, skills that researchers may still be developing.

Transparency is another important consideration. When GenAI chatbots are used to support manuscript writing, summary preparation, or data analysis, their involvement should be clearly disclosed, consistent with current publication ethics guidance [Bhavsar et al., 2025; Committee on Publication Ethics (COPE), 2025; Council of Science Editors (CSE), 2023; European Association of Science Editors (EASE), 2025; The International Association of Scientific, Technical, and Medical Publishers (STM), 2025; International Committee of Medical Journal Editors (ICMJE), 2025; Zielinski et al., 2023; Lund and Naheem, 2024]. This is especially crucial when AI-generated content influences research interpretation, patient communication, or policy briefs. Full disclosure enhances research transparency and reflects broader calls for openness in the use of digital tools in science.

A further complication is the absence of comprehensive ethical and regulatory frameworks for the use of AI in health and medical research [World Health Organization (WHO), 2025; Minssen et al., 2023; Fecher et al., 2025; World Health Organization (WHO), 2021; Sartor and Lagioia, 2020]. Although general principles for responsible AI use in healthcare are emerging, practical guidance tailored to the needs of researchers working across diverse fields remains limited. Questions around consent when using GenAI tools, transparency in GenAI-assisted outputs, and the protection of sensitive knowledge systems require more attention. Without clear guidelines and oversight mechanisms, there is a risk that researchers may misuse AI or compromise the quality and ethical integrity of their work [World Health Organization (WHO), 2025; Minssen et al., 2023; Fecher et al., 2025; World Health Organization (WHO), 2021; Sartor and Lagioia, 2020; Bouderhem, 2024]. Sharing study data with GenAI chatbots for analysis raises ethical issues related to privacy, consent, and research integrity. If the data includes sensitive or identifiable information, especially in health, social, or behavioural research, inputting it into public AI tools may breach participant confidentiality and violate informed consent agreements. Even with de-identified data, there is a risk of re-identification when data is processed in uncontrolled environments. Ethically, researchers are also responsible for ensuring the accuracy and appropriateness of analyses. Overreliance on GenAI chatbots without critical oversight may result in misinterpretation of findings, undermining the validity of the research. Additionally, the use of AI must be transparently reported to maintain integrity and accountability in the research process [World Health Organization (WHO), 2025; Minssen et al., 2023; Fecher et al., 2025; World Health Organization (WHO), 2021; Sartor and Lagioia, 2020; Bouderhem, 2024]. Legally, sharing study data with GenAI chatbots, particularly those hosted on third-party platforms, can violate data protection laws such as the General Data Protection Regulation (GDPR) or the Health Insurance Portability and Accountability Act (HIPAA), depending on jurisdiction and data type. These regulations often require data to be stored and processed in secure, regulated environments, and AI tools may not meet these standards [Sartor and Lagioia, 2020; World Health Organization (WHO), 2024; Khalid et al., 2023; Rezaeikhonakdar, 2023]. Furthermore, researchers may breach institutional review board (IRB) or ethics committee approvals if data is used in ways not previously consented to or authorized. If data is subject to contractual agreements or intellectual property rights, unauthorized use with AI tools could also lead to legal liability or disputes over data ownership and control (Pantanowitz et al., 2024; U.S Department of Health and Human Services, 2025).

Finally, at present, it is also worth acknowledging that there may be many research projects for which GenAI chatbot use offers minimal benefit or introduces unnecessary risk. In some cases, traditional human-led methods, expert consultation, or manual analysis may produce more reliable or context-sensitive results. In such contexts, deciding not to use AI may be the most scientifically rigorous and ethically responsible choice. Looking toward the future, the development of domain-specific large language models tailored to particular areas of medical research may enhance the utility, accuracy, and contextual relevance of AI support. Such models could reduce the risks presently associated with GenAI chatbots, providing more precise guidance for specialized study designs, analysis methods, or clinical and public health contexts.

In summary, although GenAI chatbots offer valuable opportunities to improve research efficiency and innovation, they come with important challenges that must be thoughtfully managed. Ensuring accurate, ethical, and context-sensitive use of AI in health and medical research requires technical competence as well as critical reflection on the social, cultural, and epistemological implications of these tools.

## 10 Conclusion

This guide highlights that prompt engineering is a vital skill for optimizing the use of GenAI chatbots across the health and medical research process. By creating prompts that are clear, structured, and aligned with the specific context of the research task, health and medical researchers can generate more accurate, useful, and ethically appropriate outputs throughout all phases of the research lifecycle, from study design and literature review to analysis, dissemination, and implementation. Still, GenAI chatbots are not replacements for critical thinking, disciplinary expertise, or methodological knowledge. Their outputs should always be considered as drafts requiring careful verification against trusted sources and thoughtful revision based on the research context. While AI tools offer potential benefits such as improving research efficiency, broadening access to information, and facilitating collaboration across disciplines, they also introduce risks, including the propagation of bias, factual inaccuracies, and oversimplified interpretations of complex issues. To use AI responsibly, researchers must approach these tools with intentionality, ethical awareness, and a commitment to reflective practice. Transparency in AI use, continual evaluation of outputs, and adherence to scholarly and professional standards are essential components of responsible integration. As digital tools continue to advance, the thoughtful application of GenAI chatbots in health and medical research holds promise not only for enhancing research quality, but also for improving the efficiency and scalability of research processes. This guide offers a foundation for engaging with GenAI chatbots in a way that upholds the principles of scientific rigour, ethical responsibility, and relevance across the health and medical research community.
