# Supplementary material for: A structured framework for effective and responsible generative artificial intelligence chatbot prompt engineering throughout the scientific process: a comprehensive guide for the health and medical researcher
Source: Front Artif Intell. 2026 Apr 16;9:1745928. doi: 10.3389/frai.2026.1745928 (PMC13130486; doi:10.3389/frai.2026.1745928)
Supplement: Supplementary file 1 [file Data_Sheet_1.DOCX]

# Supplementary File 1: Example Prompts Each Stage of the Health and Medical Research Process

The following prompts and accompanying caveats are provided as a supplement to *“From Prompt to Practice: The Health and Medical Researcher’s Comprehensive Guide to Effective and Responsible Generative Artificial Intelligence Chatbot Prompt Engineering Throughout the Scientific Process”*. These example prompts are intended to offer practical, approachable, and adaptable starting points for health and medical researchers seeking to use GenAI chatbots across various phases of the research process. Each prompt contains placeholders that can be customized to reflect a specific research topic, method, or setting. Alongside each prompt, we include a caveat to highlight key considerations in both structuring the prompt and interpreting the GenAI chatbot’s response. These caveats are intended to help researchers recognize the limitations, potential biases, and contextual gaps in GenAI chatbot-generated content, encouraging thoughtful and responsible use. Importantly, GenAI chatbot responses should not be treated as final or authoritative. They are not substitutes for critical thinking, methodological rigour, or domain expertise. Rather, they are tools to support human decision-making and spark new ideas. All outputs must be reviewed carefully, verified against credible sources, and, where necessary, discussed with subject-matter experts. As users gain experience, they are encouraged to consult the articles referenced in the guide for more advanced strategies in prompt engineering and responsible GenAI chatbot integration into health and medical research.

## 7.2 Research Question Development and Study Design: Example Prompts

1. **“Help me transform my broad research idea on [disease/intervention] into a specific, answerable research question using the PICOT framework.”**
   *Caveat(s):* Be sure to provide enough detail in your initial idea for the AI chatbot to generate a meaningful response. The model may suggest questions that sound plausible but overlook context-specific factors such as feasibility, population-specific nuances, or ethical constraints. Always refine the AI chatbot-suggested question using your disciplinary expertise and check for alignment with your research goals.
2. **“Suggest appropriate study designs for investigating [intervention] in [population]. Please explain the strengths and limitations of each.”**
   *Caveat(s):* The AI chatbot may offer textbook-style summaries of study designs without tailoring them to the specific context you are working in. Avoid over-relying on these suggestions; review them critically and assess whether each design is appropriate given your ethical, logistical, and resource constraints.
3. **“Based on the aim to evaluate [intervention] for [condition], what would be suitable primary and secondary outcomes?”**
   *Caveat(s):* The AI chatbot might suggest outcomes that are either too vague or overly clinical and not aligned with patient-relevant or stakeholder-informed priorities. Use its suggestions as a starting point but validate them against the literature, clinical guidelines, or expert consensus documents.
4. **“Can you outline a conceptual framework linking [exposure] to [outcome] in the context of [setting]?”**
   *Caveat(s):* Conceptual frameworks generated by the AI chatbot may oversimplify complex causal pathways or exclude key variables. Ensure that the logic reflects both theoretical grounding and real-world relevance. You may need to adapt or expand the framework based on current literature and contextual understanding.
5. **“I am interested in studying [health behavior] in [population]. What are some ethical considerations and possible study designs I should explore?”**
   *Caveat(s):* AI chatbot suggestions may not fully capture culturally sensitive or legally mandated ethical considerations. Use its responses to prompt deeper reflection, but consult ethics review board guidelines and local norms when finalizing your study design and procedures.
6. **“What are some advantages and challenges of using a mixed methods design for studying [topic]?”**
   *Caveat(s):* The AI chatbot may present general pros and cons without referencing your specific research context. Be cautious about using overly generic rationales. Consider your own capacity to conduct both qualitative and quantitative components with methodological rigour.
7. **“Based on the problem of [clinical issue], can you propose a logic model that links inputs, activities, outputs, and outcomes?”**
   *Caveat(s):* Logic models generated by AI chatbot may appear complete but can miss key assumptions, context-specific barriers, or unintended outcomes. Use the model as a draft, but ensure it reflects the realities of your implementation setting through consultation with stakeholders or collaborators.
8. **“Suggest potential confounding variables I should consider when studying [exposure] and [outcome].”**
   *Caveat(s):* The AI chatbot may omit important confounders or suggest irrelevant ones depending on how specific your prompt is. Be sure to cross-reference the suggestions with epidemiological literature and consider adjusting for confounders based on both theoretical and empirical evidence.
9. **“Can you list feasibility concerns for conducting a randomized controlled trial on [intervention] in [setting]?”**
   *Caveat(s):* AI chatbot-generated feasibility issues may not fully reflect real-world logistical, financial, or regulatory challenges. Ensure that feasibility assessments incorporate local knowledge, institutional policies, and practical experience from similar studies.
10. **“Explain the key differences between a pragmatic trial and an explanatory trial in the context of [study topic].”**
    *Caveat(s):* AI chatbot may provide an accurate general distinction but not tailor it to your specific study goals. Pragmatic vs. explanatory elements exist along a continuum, so verify that the distinctions align with accepted frameworks such as PRECIS-2 and adapt them based on the intended purpose of your trial.

## 7.3 Literature Searches for Background and Narrative Reviews: Example Prompts

1. **“Generate a list of keywords and synonyms related to [health condition/intervention] for a narrative literature review.”**
   *Caveat(s):* The AI chatbot may generate outdated, overly broad, or irrelevant keywords. Always cross-check with controlled vocabularies (e.g., MeSH), recent review papers, and expert input to ensure comprehensive coverage.
2. **“What are some key concepts and emerging terms in the literature on [topic] that I should be aware of?”**
   *Caveat(s):* The AI chatbot may not distinguish between well-established and speculative terms. Use its output as a brainstorming aid, but verify suggested terms using current peer-reviewed literature or discipline-specific databases.
3. **“Summarize recent developments in [field/topic] over the past 5 years using only peer-reviewed sources.”**
   *Caveat(s):* AI chatbot chatbots do not have real-time access to databases and may generate outdated or hallucinated content. Always verify summaries against current journal articles, systematic reviews, or bibliometric tools.
4. **“Explain the difference between [term 1] and [term 2] as used in the context of [field].”**
   *Caveat(s):* Definitions may be context-dependent, and the AI chatbot may not reflect discipline-specific nuances. Consult authoritative sources or domain experts if clarity is critical for your work.
5. **“List at least five related topics or subtopics to [research area] that I might consider including in my literature review.”**
   *Caveat(s):* AI chatbot-generated lists may omit emerging subfields or overly focus on mainstream areas. Use them to prompt further exploration, but validate through exploratory database searches or scoping reviews.
6. **“Provide a plain-language explanation of [theory/concept] relevant to my research on [topic].”**
   *Caveat(s):* While helpful for accessibility, the AI chatbot may oversimplify or misrepresent theoretical frameworks. Review original sources or theory-based texts to ensure conceptual accuracy.
7. **“Summarize the key challenges and debates surrounding [issue] in current medical literature.”**
   *Caveat(s):* The AI chatbot may produce summaries that are too generic or miss key controversies. Use this as a starting point, then refine your understanding through critical reading of commentaries or editorials.
8. **“I found a paper on [topic]; can you help me understand the abstract and identify its relevance to my project?”**
   *Caveat(s):* The AI chatbot may misinterpret study objectives or findings. Always read the full paper and consult a topic expert before deciding on inclusion or exclusion.
9. **“Provide an overview of the most common interventions studied in relation to [condition].”**
   *Caveat(s):* AI chatbot outputs may disproportionately reflect well-known interventions and exclude emerging or context-specific practices. Use this prompt to generate ideas, but confirm through systematic search strategies.
10. **“List potential biases or gaps that may appear in narrative reviews on [topic].”**
    *Caveat(s):* The AI chatbot might list general risks (e.g., selection bias) but fail to link them to the specific topic. You may need to tailor and elaborate based on your review scope, sources, and audience.

## 7.4 Reviews with Systematic Search Components: Example Prompts

1. **“Help me develop a preliminary research question for a systematic review on [intervention] for [condition].”**
   *Caveat(s):* AI chatbot-generated questions may not meet the specificity requirements for systematic reviews. Always revise using the PICOT framework and consult guidance documents such as PRISMA-P.
2. **“Suggest MeSH and Emtree terms related to [topic] for a comprehensive search strategy.”**
   *Caveat(s):* AI chatbots do not have real-time access to MeSH or Emtree vocabularies. Validate all suggested terms using the official thesauri or by consulting an information specialist.
3. **“List possible Boolean search strings for combining [population], [intervention], and [outcome].”**
   *Caveat(s):* AI chatbot may format Boolean operators incorrectly or omit parentheses and truncation symbols. Double-check all search logic and syntax before use in databases.
4. **“What are some common databases to search for literature on [topic] in public health?”**
   *Caveat(s):* The chatbot might suggest general databases while omitting specialized or regional sources. Cross-reference with librarian recommendations or published reviews.
5. **“Recommend ways to structure inclusion and exclusion criteria for a scoping review on [topic].”**
   *Caveat(s):* Outputs may lack nuance or fail to reflect review objectives. Use as inspiration but refine with reference to JBI or PRISMA-ScR guidance.
6. **“Generate alternative search terms for [intervention] used in various clinical contexts.”**
   *Caveat(s):* AI chatbot may suggest terms used inconsistently or historically. Validate terms by reviewing key studies or running preliminary searches to assess term relevance.
7. **“Can you help draft a basic search strategy in MEDLINE for [condition] and [treatment]?”**
   *Caveat(s):* AI chatbot may produce search strings that appear correct but include fabricated or irrelevant terms. Always test outputs in actual databases and revise as needed.
8. **“What are some ways to organize extracted data from studies on [topic] in a tabular format?”**
   *Caveat(s):* Tables suggested may be overly simplistic or miss key fields such as study setting, funding source, or outcome measure type. Customize to suit review objectives.
9. **“List potential sources of grey literature relevant to [research question].”**
   *Caveat(s):* The AI chatbot may suggest generic repositories and omit discipline-specific sources (e.g., clinical trial registries, policy databases). Confirm through grey literature search guides or expert consultation.
10. **“Explain the difference between a scoping review and a systematic review in the context of [topic].”**
    *Caveat(s):* The distinction may be described accurately but not applied correctly to your topic. Use the explanation as a foundation, but refer to methodological guidance (e.g., JBI Manual) for review selection.

## 7.5 Research Methodology, Reporting Guidelines, and Critical Appraisal: Example Prompts

1. **“Identify which reporting guidelines are most appropriate for a [study design] investigating [intervention] in [population].”**
   *Caveat(s):* AI chatbot may suggest well-known guidelines but overlook more specific extensions or nuanced scenarios. Always cross-check with the EQUATOR Network or relevant methodological literature to ensure appropriateness.
2. **“Explain the purpose and main sections of the CONSORT checklist in accessible terms.”**
   *Caveat(s):* While the AI chatbot can simplify technical language, it may omit important nuances or misrepresent elements. Review the actual CONSORT statement to ensure nothing critical is lost in translation.
3. **“Suggest a critical appraisal tool suitable for a cross-sectional study on [health behavior] and describe its key components.”**
   *Caveat(s):* The AI chatbot might recommend inappropriate or outdated tools. Validate the suggested tool’s relevance through academic resources or the tool’s publication or website.
4. **“What are the main reporting standards for a qualitative study using thematic analysis?”**
   *Caveat(s):* AI chatbot may confuse reporting standards with methodological frameworks or offer general guidelines without tailoring to qualitative traditions. Confirm recommendations with resources, such as COREQ or SRQR.
5. **“Compare AGREE and RIGHT reporting guidelines for systematic reviews.”**
   *Caveat(s):* Comparisons may be superficial and overlook context-specific applicability. Supplement AI chatbot-generated comparisons with the official checklists and guidelines.
6. **“Explain how the STROBE checklist applies to an observational study examining [exposure] and [outcome].”**
   *Caveat(s):* AI chatbot may provide a generic interpretation that fails to address design-specific elements (e.g., cohort vs. cross-sectional). Always consult the full STROBE checklist for application.
7. **“Explain the difference between internal validity and external validity in clinical trials.”**
   *Caveat(s):* Definitions may be accurate but oversimplified. The AI chatbot may not relate them to specific trial features such as eligibility criteria or setting. Use the output as a teaching tool, not final analysis.
8. **“List at least three commonly used quality appraisal tools and describe when each should be used.”**
   *Caveat(s):* AI chatbot might conflate quality appraisal tools with risk of bias tools or suggest tools without specifying study design relevance. Ensure alignment with the research method in question.
9. **“How can I ensure transparency and reproducibility when reporting findings from a mixed methods study?”**
   *Caveat(s):* Suggestions may focus on general best practices and overlook discipline-specific guidance (e.g., MMAT or GRAMMS). Always adapt advice to your field and journal requirements.
10. **“Help me understand the concept of risk of bias in randomized trials and where it is addressed in the CONSORT checklist.”**
    *Caveat(s):* AI chatbot may not provide a complete picture of how risk of bias is reported across different trial stages. Cross-check with Cochrane RoB tools and CONSORT explanations to ensure thorough coverage.

## 7.6 Quantitative Analysis: Example Prompts

1. **“Explain the difference between [test 1] and [test 2] and when each should be used in analyzing [type of data].”**
   *Caveat(s):* The AI chatbot may provide oversimplified criteria (e.g., “use this when data is not normal”) and fail to include assumptions, limitations, or alternatives. Confirm with a statistical textbook or statistician.
2. **“Suggest appropriate statistical tests for analyzing the effect of [intervention] on [outcome] in a [study design] with [sample size] participants.”**
   *Caveat(s):* The AI chatbot may suggest a test without context-specific reasoning (e.g., ignoring clustering, non-normality, or repeated measures). Evaluate assumptions and consult a statistician if unsure.
3. **“Generate R code to conduct a [statistical test] on a dataset examining [exposure] and [outcome].”**
   *Caveat(s):* Even when prompted to verify, AI chatbot-generated code may contain syntax errors, use deprecated functions, or rely on incorrect assumptions. Always test code on sample data and consult documentation.
4. **“I am planning to analyze longitudinal data on [condition]. What models should I consider, and what are their assumptions?”**
   *Caveat(s):* The model may recommend inappropriate techniques (e.g., repeated-measures ANOVA when mixed models are better). Use this for orientation only; confirm your analytic plan with a methodologist.
5. **“Explain what a generalized linear model is and how it might be applied to non-normally distributed data on [topic].”**
   *Caveat(s):* AI chatbot may skip over model specification details (e.g., link functions, variance structures). Use the explanation as an introduction, but seek applied examples in peer-reviewed sources.
6. **“Provide a step-by-step explanation of how to check for missing data patterns and manage missing values in [software].”**
   *Caveat(s):* Instructions may omit important diagnostics (e.g., Little’s MCAR test) or recommend methods without justification. Always refer to up-to-date tutorials and statistical guidance.
7. **“What are common statistical errors to avoid when interpreting p-values in a study on [intervention]?”**
   *Caveat(s):* The AI chatbot may highlight common misconceptions (e.g., p ≠ probability of truth) but not cover context-specific misinterpretations (e.g., multiple testing). Use it as a discussion prompt, not final advice.
8. **“Suggest methods to adjust for confounding in a regression analysis exploring the relationship between [variable 1] and [variable 2].”**
   *Caveat(s):* AI chatbot may suggest common techniques (e.g., multivariable regression) but not address issues such as multicollinearity or residual confounding. Validate against method papers or a statistician.
9. **“Generate Python code for visualizing [type of data].”**
   *Caveat(s):* AI chatbot may produce visually appealing but inefficient or incorrect code. Always test visualizations using test data and interpret graphics in the context of your research question.
10. **“List some assumptions of [statistical method] and explain how violating them could impact results.”**
    *Caveat(s):* AI chatbot may list assumptions incompletely or incorrectly (e.g., confusing normality of residuals with normality of variables). Always double-check using authoritative statistical resources.

## 7.7 Qualitative Research: Example Prompts

1. **“Help me generate open-ended interview questions for a qualitative study exploring [experience] among [population].”**
   *Caveat(s):* AI chatbot-generated questions may sound generic or lack cultural sensitivity. Always adapt the phrasing to reflect your study context, theoretical framework, and participant group.
2. **“Suggest a coding framework for analyzing qualitative data from focus groups about [health topic].”**
   *Caveat(s):* The AI chatbot may suggest broad or off-the-shelf frameworks that do not align with your epistemological stance. Ensure your choice of framework is appropriate for your methodological approach.
3. **“What are the key features of thematic analysis, and how might it apply to my data on [subject]?”**
   *Caveat(s):* The response may summarize one type of thematic analysis without acknowledging alternative approaches. Confirm that the version aligns with your research aims and reflexivity practices.
4. **“Explain the differences between grounded theory and framework analysis in the context of analyzing [type of data].”**
   *Caveat(s):* The AI chatbot might oversimplify differences, particularly in relation to epistemological underpinnings or iterative design elements. Supplement with methodological literature to deepen understanding.
5. **“Generate example codes that could be applied to interview transcripts related to [experience].”**
   *Caveat(s):* Suggested codes may be superficial or prematurely interpretive. Ensure they are grounded in actual data and refined through iterative coding by the researcher.
6. **“How can I ensure reflexivity when conducting qualitative research on [sensitive topic]?”**
   *Caveat(s):* AI chatbot may give general advice (e.g., “keep a reflexive journal”) without discussing power dynamics, positionality, or context. Integrate these suggestions with qualitative research methodological literature.
7. **“Suggest ways to present qualitative findings in a manuscript, including how to incorporate participant quotes.”**
   *Caveat(s):* The structure may follow basic conventions but miss field-specific expectations (e.g., for grounded theory or narrative inquiry). Tailor reporting to your chosen methodology and journal guidelines.
8. **“What are some ethical considerations when collecting qualitative data from [vulnerable group]?”**
   *Caveat(s):* AI chatbot may omit important ethical concerns such as trauma-informed interviewing or participatory research principles. Always consult ethical guidelines specific to your context and participant group.
9. **“Help me generate a memo summarizing analytic reflections after a coding session on [topic].”***Caveat(s):* AI-generated memos may mimic the structure of analytic writing but lack insight grounded in your actual data engagement. Ensure that any memos used for analysis authentically reflect your interpretations, observations, and evolving insights—not just generic summaries.
10. **“Describe how to develop a coding tree based on an initial set of codes from interviews on [research question].”**
    *Caveat(s):* Coding trees suggested by AI chatbot may reflect hierarchical models that do not fit all analytical frameworks. Align structure with your methodological approach (e.g., inductive vs. deductive coding).

## 7.8 Writing and Dissemination: Example Prompts

1. **“Draft a plain-language summary of findings from a published study on [topic] that would be appropriate for [audience type]. I will revise for accuracy.”**
   *Caveat(s):* The AI chatbot may oversimplify or misstate findings. Always fact-check and adjust tone, ensuring clarity without distortion of scientific meaning.
2. **“Help me write a structured abstract for a conference submission on [study design] investigating [intervention] in [population].”**
   *Caveat(s):* AI chatbot may produce a generic format that misses field-specific conventions or word limits. Edit for conciseness, accuracy, and alignment with conference guidelines.
3. **“Suggest a social media post summarizing the main findings of a study on [condition] for use on [platform]”**
   *Caveat(s):* AI chatbot-generated posts may use buzzwords or simplified claims that risk misinterpretation. Carefully refine for tone, brevity, and accuracy, especially for high-visibility platforms.
4. **“What are some key elements of an effective press release for a publication on [topic]?”**
   *Caveat(s):* The AI chatbot may suggest generic elements that do not meet journal or institutional requirements. Verify formatting and messaging with your communications team if possible.
5. **“Generate a draft cover letter for submitting a manuscript on [research focus] to [journal name].”**
   *Caveat(s):* Cover letters often require tailored messaging and should highlight study fit with the journal. Treat AI chatbot output as a framework, not a final draft.
6. **“Convert this technical paragraph on [intervention] into a lay summary suitable for patient education materials.”**
   *Caveat(s):* AI chatbot may introduce ambiguity or misrepresent clinical nuance. Ensure health literacy standards are met and verify with professionals experienced in patient communication.
7. **“Help me develop a short [platform] summary post that highlights the policy implications of our research on [issue].”**
   *Caveat(s):* Outputs may overstate significance or omit essential qualifiers. Carefully verify claims and ensure accuracy with actual findings and implications.
8. **“Translate this summary of findings on [topic] into [language] for public communication.”**
   *Caveat(s):* Machine-generated translations may miss cultural nuance or idiomatic meaning. Always confirm with a fluent, domain-competent speaker.
9. **“Suggest ways to adapt the tone and style of this manuscript section for a non-specialist audience.”**
   *Caveat(s):* The AI chatbot may not differentiate between audiences (e.g., policymakers vs. patients). Specify your audience clearly and edit accordingly.
10. **“Help me generate bullet points summarizing the clinical relevance of [study finding] for use in a visual abstract.”**
    *Caveat(s):* AI chatbot may suggest overgeneralized or exaggerated claims. Ensure that each bullet point accurately reflects the study’s actual results and limitations.

## 7.9 Implementation: Example Prompts

1. **“Suggest potential implementation strategies for integrating [evidence-based intervention] into [clinical setting].”**
   *Caveat(s):* AI chatbot may suggest generic or idealized strategies without considering local constraints (e.g., staffing, policies, funding). Use output for brainstorming only and refine based on feasibility assessments and stakeholder input.
2. **“What are common barriers and facilitators to implementing [type of intervention] in [population] or [geographic location]?”**
   *Caveat(s):* AI chatbot may generate a list of common issues (e.g., “lack of training,” “resource limitations”) but overlook population-specific factors such as cultural attitudes, infrastructure, or regulatory environments. Validate with implementation science literature and local data.
3. **“Draft a logic model outlining inputs, activities, outputs, and outcomes for a project aiming to implement [intervention] in [setting].”**
   *Caveat(s):* AI chatbot-generated logic models may omit critical assumptions or external influences and can appear deceptively comprehensive. Treat them as drafts and refine in collaboration with stakeholders and implementation experts.
4. **“Explain how the CFIR framework could be applied to identify challenges in implementing [program/policy] in [context].”**
   *Caveat(s):* The AI chatbot may summarize CFIR domains superficially without tailoring to your intervention or setting. You must adapt each domain (e.g., inner setting, outer setting) to your specific implementation context.
5. **“Help me brainstorm ways to tailor a [health service innovation] to rural or low-resource settings.”**
   *Caveat(s):* Suggestions may rely on assumptions from high-income settings or fail to reflect real-world resource limitations. Use output as a prompt for deeper exploration through participatory or community-engaged approaches.
6. **“Suggest components of a training module for healthcare providers on implementing [guideline or practice change].”**
   *Caveat(s):* AI chatbot may propose standard training components (e.g., “interactive sessions,” “handouts”) without aligning with adult learning principles or provider workflow. Validate with educational design principles and provider feedback.
7. **“What are the RE-AIM framework domains, and how can they be used to evaluate the implementation of [initiative]?”**
   *Caveat(s):* The AI chatbot may define RE-AIM domains correctly but fail to show how they relate to your initiative. Supplement with case studies and tailor the application of each domain to your goals and context.
8. **“Create a draft implementation timeline for a [type of intervention] roll-out in [healthcare context].”**
   *Caveat(s):* AI chatbot timelines may not account for real-world delays, stakeholder readiness, or iterative phases of rollout. Review and revise with consideration of regulatory, logistical, and organizational timelines.
9. **“Generate talking points to engage stakeholders in a discussion about implementing [intervention] in [community or institution].”**
   *Caveat(s):* Talking points may sound persuasive but may lack cultural appropriateness, relevance, or alignment with stakeholder priorities. Ensure they are co-developed or reviewed with actual stakeholders.
10. **“Suggest approaches for adapting [intervention] to meet cultural or contextual needs in [specific population], while maintaining fidelity to the evidence.”**
    *Caveat(s):* AI chatbot may not distinguish between surface-level and deep-level cultural adaptations or may offer vague strategies (e.g., “consider language differences”). Refer to implementation science frameworks (e.g., ADAPT-ITT, FRAME) for structured adaptation processes.
